# Acute Respiratory Distress Syndrome: A Rare Manifestation of Rhinovirus Infection

**DOI:** 10.7759/cureus.43484

**Published:** 2023-08-14

**Authors:** Alexander T Phan, Henrik Ghantarchyan, Viet-Tien Nguyen, Mufadda Hasan

**Affiliations:** 1 Internal Medicine, Arrowhead Regional Medical Center, Colton, USA; 2 Pulmonary and Critical Care Medicine, Arrowhead Regional Medical Center, Colton, USA

**Keywords:** acute respiratory distress syndrome [ards], ards, pulmnology, pneumonia, immunosuppression, acute respiratory distress syndrome, human rhinovirus

## Abstract

Human rhinovirus (HRV) is a common cause of respiratory infections. HRV-related lower respiratory tract infections, including community-acquired pneumonia, are seldom seen in the clinical setting, and progression to acute respiratory distress syndrome (ARDS) is even rarer. We report on a case of a young immunosuppressed host who presented to the hospital for respiratory distress. She was diagnosed with HRV-related pneumonia, which rapidly progressed to ARDS based on clinical examination. After orotracheal intubation and mechanical ventilation with a low tidal volume strategy, she made a rapid recovery. This case highlights the importance of understanding that HRV may be an etiology of community-acquired pneumonia in immunosuppressed hosts and that ARDS may be a complication of this infection. Rapid recognition and clinical suspicion are important to the care of these patients, as ARDS has a high mortality rate.

## Introduction

Human rhinoviruses (HRVs) are of the genus Enterovirus and family Picornaviridae. HRVs are separated into groups A, B, and C based on genetic structure. HRVs are responsible for the majority of upper respiratory tract infections and are also associated with sinusitis and otitis media. The severity of HRV pulmonary infections is related to the amount of inflammatory cytokines produced, namely interleukin-1 (IL-1), IL-6, and IL-8. At this time, evidence also suggests that they are linked to lower respiratory tract infections, especially in patients with pulmonary disorders, of old age, and who are immunosuppressed. Since no definitive antiviral treatment is available for HRV-related infections, supportive therapies remain the mainstay of management [1].

HRVs are primarily transmitted via contact or aerosolization, with viruses being taken up through macropinocytosis and endocytosis [1]. In cases of severe lower respiratory tract infections, diffuse alveolar damage may ensue, leading to acute respiratory distress syndrome (ARDS) [2]. ARDS is defined by the Berlin Criteria: (1) bilateral opacities on chest imaging not fully explained by effusions, lobar/lung collapse, or nodules; (2) respiratory failure not explained by cardiac failure of volume overload; (3) occurrence within one week of known clinical insult or new or worsening respiratory symptoms; and (4) impaired oxygenation with PaO2/FiO2 ratio of 300 or less. Rhinovirus is a rare cause, only reported on a case report level, and more commonly affects immunosuppressed hosts [2-3]. The mortality associated with ARDS has been cited to be from 38.5% to 58%; as such, raising awareness of this manifestation of HRV infections is of the utmost importance [4-5]. Here, we present the case of a 33-year-old female who presented with acute hypoxemic respiratory failure with rapidly increasing oxygen requirements and diffuse pulmonary opacities. HRV was detected in a nasopharyngeal sample and ARDS mechanical ventilation protocol was initiated.

## Case presentation

A 33-year-old female with a past medical history of autoimmune hepatitis status post orthotopic liver transplantation in 1999, Crohn's disease status post colectomy in 2019, and asthma presented to the hospital complaining of progressive dyspnea over the past day that was unrelieved by nebulized albuterol. Five days prior to her hospital presentation, she developed a sore throat, dry cough, and nausea. She was treated at an urgent care facility four days prior to hospital presentation with amoxicillin due to otitis media; however, her cough and sore throat did not improve. She admitted to subjective fevers, chills, and rhinorrhea that started two days prior to the hospital presentation. She denied sick contacts. En route to the hospital, she was placed on 15 liters (L) of supplemental oxygen via a non-rebreather mask due to increased work of breathing and suspected hypoxia. Her home medications included oral tacrolimus 2mg twice daily and oral budesonide 3mg twice daily.

**Figure 1:**
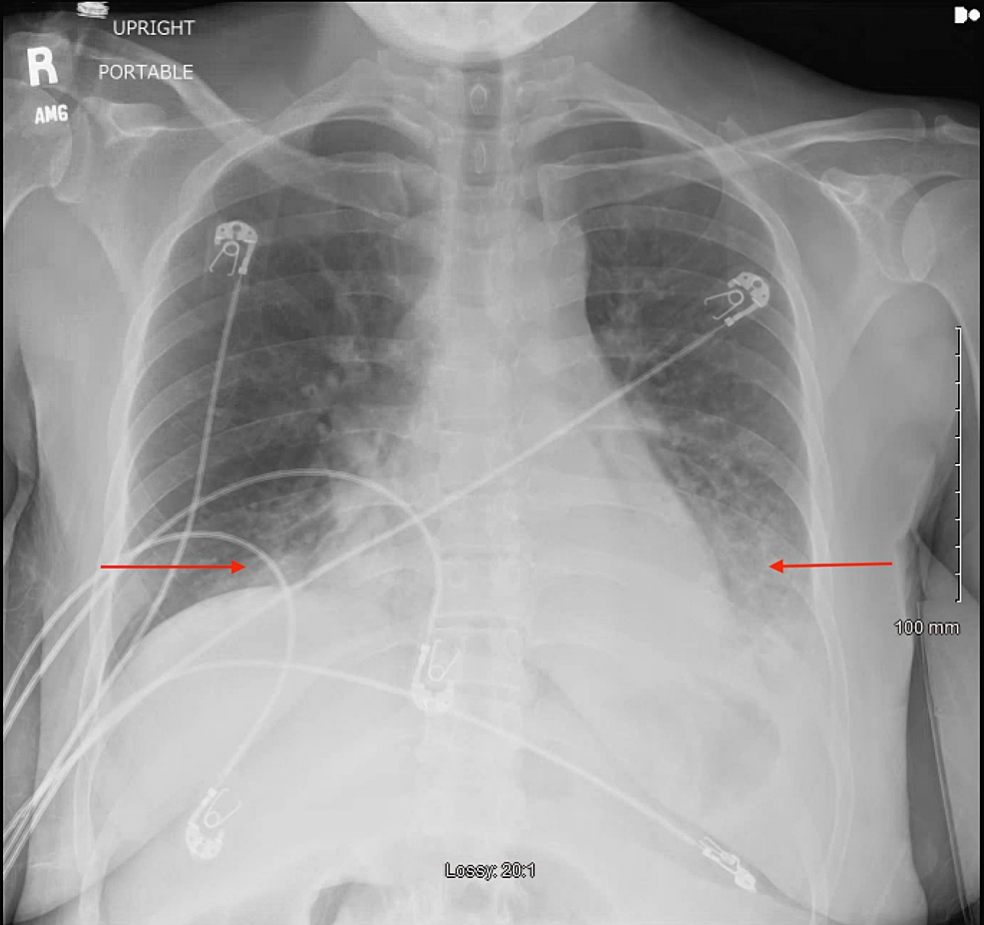
Anterior-posterior chest radiograph demonstrating bilateral opacifications of the lower lung fields (red arrows).

**Figure 2:**
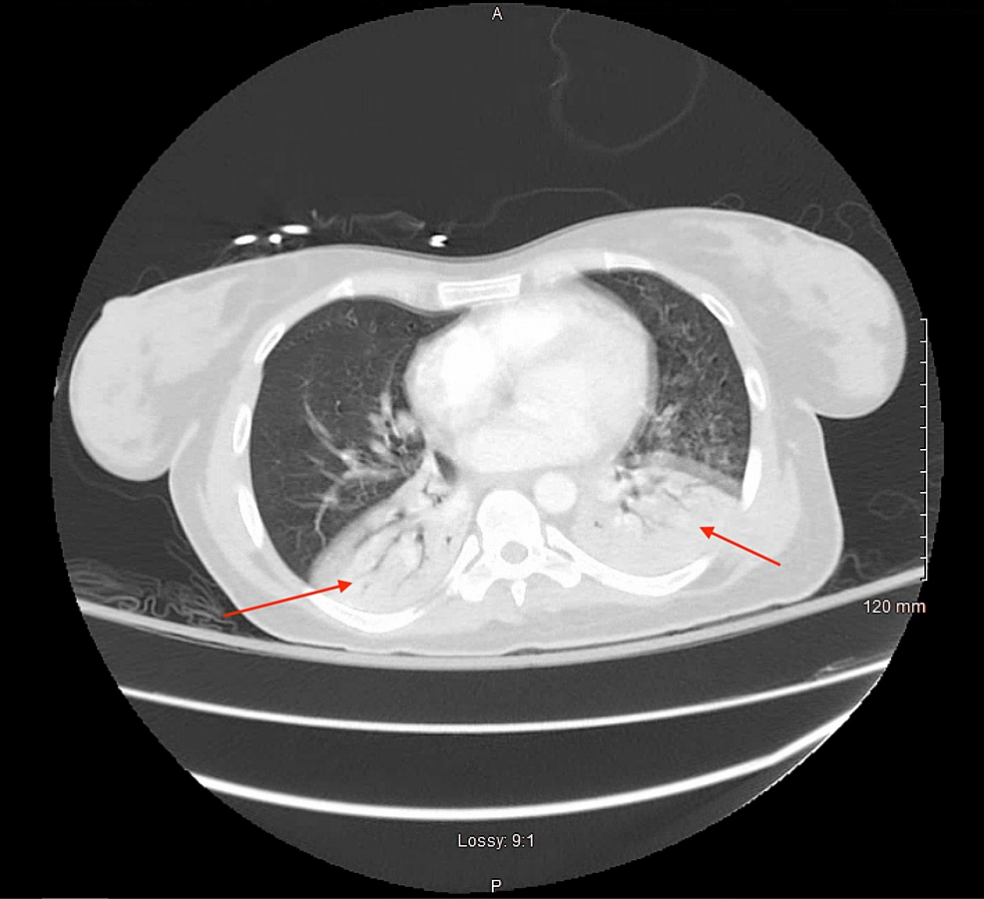
A computed tomography angiogram of the chest was obtained demonstrating bilateral lower lobe consolidations with air bronchograms (red arrows)

Initial vital signs included a blood pressure of 152/88 mmHg, pulse rate of 121, respiratory rate of 49, temperature of 99.1 °F, and oxygen saturation of 98% on high flow nasal cannula with settings of 60L flow rate and 100% fraction of inspired oxygen (FiO2). Physical examination was notable for an ill-appearing female patient in acute distress, tachycardia, tachypnea, accessory muscle use, and increased work of breathing. A chest radiograph demonstrated bilateral opacifications in the bilateral lower lung fields (Figure 1). Initial laboratory investigations demonstrated bandemia without leukocytosis, normocytic anemia, high anion gap metabolic acidosis with concomitant respiratory alkalosis on the aforementioned supplemental oxygen settings, elevated lactate, elevated serum creatinine, and unremarkable urinalysis (Table 1). A nasopharyngeal swab sample was obtained for a respiratory panel (multiplex amplified nucleic acid testing) demonstrating a positive result for human rhinovirus. A computed tomography (CT) angiogram of the chest was obtained, demonstrating bilateral lower lobe consolidations with air bronchograms and no evidence of pulmonary embolism (Figure 2).

**Table 1:** Initial laboratory investigations demonstrating bandemia without leukocytosis, normocytic anemia, high anion gap metabolic acidosis with concomitant respiratory alkalosis, elevated lactate, elevated serum creatinine. BUN: blood urea nitrogen, MCV: mean corpuscular volume, μL: microliter, g: gram, mg: milligram dL: deciliter, mEq: milliequivalent, U: unit, L: liter, mmol: millimole, mmHg = millimeters of mercury

Laboratory Test	Reference Values	Measured Values
White Blood Cells (cells/μL)	4,300-11,100	9,600
Band Neutrophils (%)	0	21
Hemoglobin (g/dL)	11.5-15.5	11.4
Hematocrit (%)	36-46	35
Platelet (cells/μL)	120,000-360,000	164,000
Mean Corpuscular Volume (fL)	80-100	87
Sodium (mEq/L)	135-148	135
Potassium (mEq/L)	3.5-5.5	4
Chloride (mEq/L)	98-110	99
Bicarbonate (mmol/L)	24-34	17
BUN (mg/dL)	8-20	35
Creatinine (mg/dL)	0.5-1.5	1.48
Calcium (mg/dL)	8.5-10.5	10.4
Phosphorous (mg/dL)	2.4-4.4	2.4
Albumin (g/dL)	3.5-4.9	3.6
Arterial pH	7.35-7.45	7.43
Arterial Carbon Dioxide (mmHg)	35-45	29
Arterial Oxygen (mmHg)	75-100	79
Arterial Carboxyhemoglobin (%)	0-1.5	0.30%
Arterial Lactate (mmol/L)	0-2	2.39

**Figure 3:**
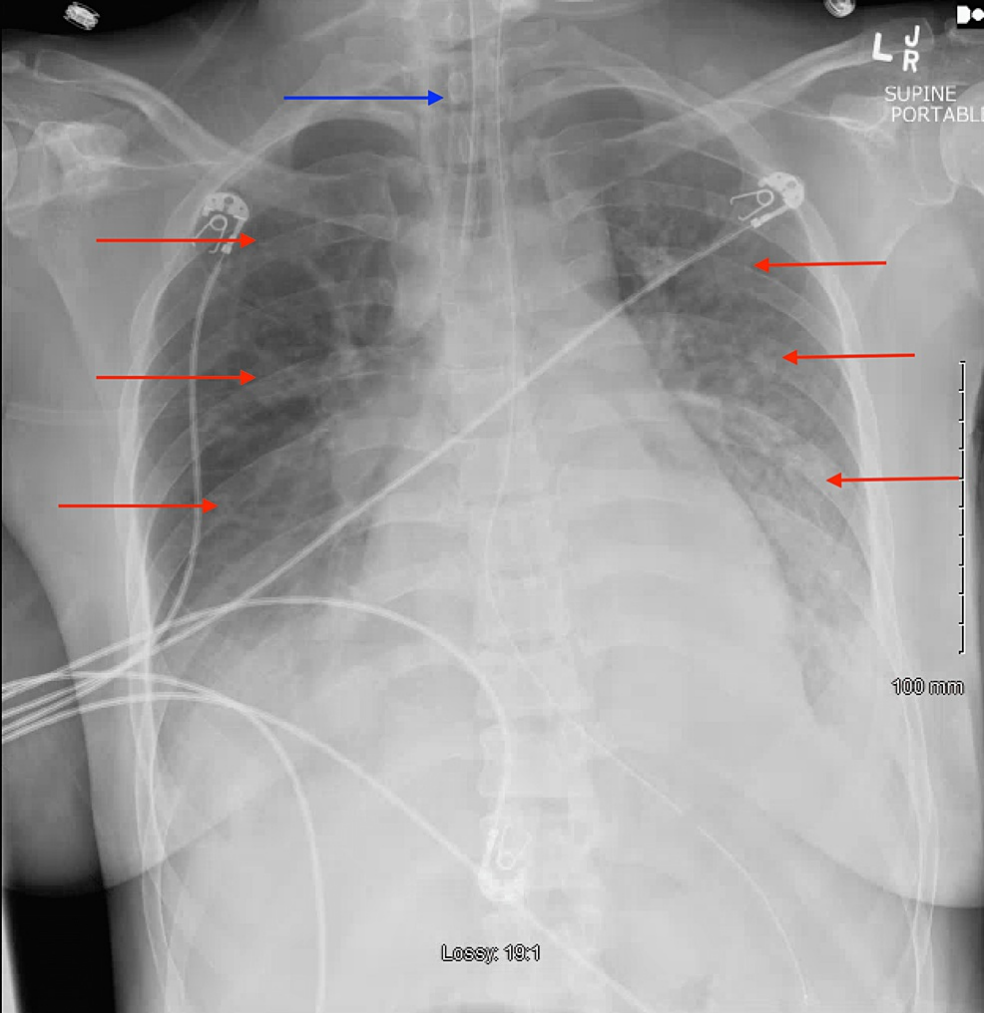
Anterior-posterior chest radiograph demonstrating interval intubation (blue arrow) and diffuse bilateral opacifications (red arrows)

At this time, through shared decision-making, the patient was electively orotracheally intubated due to the high risk of respiratory decompensation and sedated for comfort. A post-intubation chest radiograph demonstrated diffuse bilateral pulmonary opacifications (Figure 3). Due to a partial pressure of arterial oxygen (PaO2) to a fraction of inspired oxygen (FiO2) ratio of 79 (Table 1), diffuse bilateral opacifications on chest imaging, rapid onset of illness, and non-cardiogenic/ volume overload etiology of respiratory failure and a low tidal volume, lung protective ventilation strategy was initiated for a diagnosis of ARDS. Mechanical ventilator settings were initiated as follows: pressure control/assist control mode, peak inspiratory pressure (PIP) of 27 centimeters of water (cm H2O), peak end-expiratory pressure (PEEP) of 8 cm H2O, respiratory rate of 14, and FiO2 of 100%. A transthoracic echocardiogram was performed, demonstrating a left ventricular ejection fraction of 65% without evidence of diastolic heart failure. The patient was initiated on broad-spectrum antibiotics: intravenous (IV) vancomycin 20 mg/kg, IV cefepime 2g every 12 hours, IV metronidazole 500mg every eight hours, and IV azithromycin 500mg once daily.

**Figure 4:**
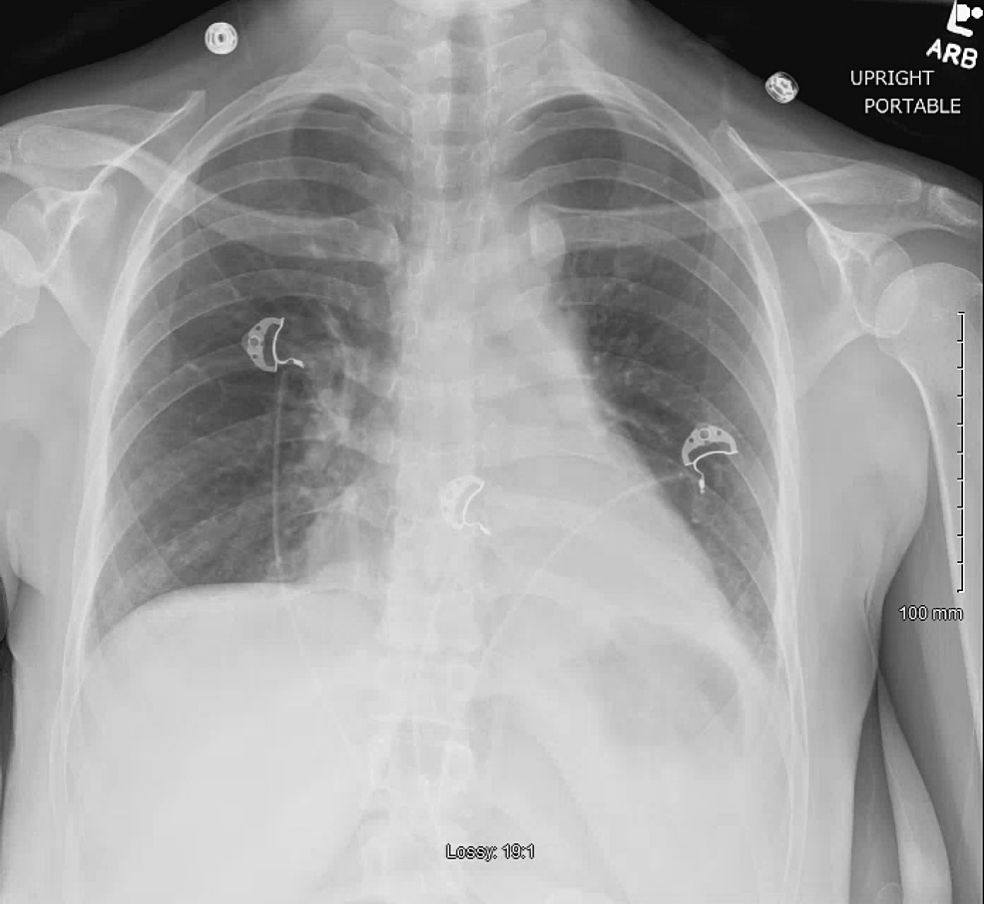
Anterior-posterior chest radiograph status post extubation

Urinary legionella antigen and streptococcus pneumoniae antigen tests were negative, blood cultures did not demonstrate evidence of bacteremia, and respiratory cultures of endotracheal aspirates did not demonstrate evidence of bacterial infection. A serum procalcitonin level was obtained and was 0.1 nanograms per milliliter (ng/mL) (N < 0.08 ng/mL). The patient’s acute kidney injury resolved with intermittent boluses of 500 mL lactated ringer solution. Antibiotics were discontinued on Day 3 of hospitalization due to a lack of evidence suggesting a bacterial source and clinical improvement. The patient was extubated on Day 3 of hospitalization due to improvement in oxygenation (PaO2 to FiO2 ratio >300) on minimal ventilator settings (respiratory rate of 12, FiO2 of 30%, PIP of 18 cm H2O, and PEEP of 5 cm H2O). The arterial blood gas prior to extubation demonstrated a pH of 7.37, arterial carbon dioxide of 30 mmHg, arterial oxygen of 101 mmHg, and arterial bicarbonate of 17 mmol/L. A post-extubation chest radiograph can be seen in Figure 4. She was discharged on Day 5 of hospitalization after she tolerated an oral diet and was well enough to ambulate on ambient air. On follow-up, the patient is alive and well, and has not required hospitalization since discharge.

## Discussion

Our case centers on the clinical course of an immunosuppressed female patient who presented with respiratory failure. The initial differential diagnosis included asthma exacerbation, bacterial pneumonia, viral pneumonia, organizing pneumonia, and pulmonary embolism. To narrow down the differential diagnosis, we ruled out pulmonary embolism and asthma exacerbation based on computed tomography and physical examination findings, respectively. Bacterial infection was ruled out based on our infectious work-up, which included endotracheal aspirate cultures, urinary streptococcus pneumoniae antigen, and urinary legionella antigen. Nasopharyngeal swab testing was negative for coronaviruses, human metapneumovirus, parainfluenza viruses, influenza A, influenza B, respiratory syncytial virus, Bordetella pertussis, Bordetella parapertussis, Chlamydia pneumoniae, and Mycoplasma pneumoniae. Due to a positive rhinovirus result on nasopharyngeal swab testing and the negative testing for other infectious etiologies, rhinovirus pneumonia complicated by ARDS became the leading diagnosis.

HRVs do not commonly affect the lower respiratory tract in adults, with only 5% of cases leading to community-acquired pneumonia [1]. HRV-related pneumonia is more commonly observed in children, likely due to their immature immune systems. Similarly, immunosuppressed adults are also at increased risk of HRV pneumonia [2]. Therefore, it is essential to be particularly vigilant with immunosuppressed patients, as HRV-related infections can be especially severe in this population. In a study by Jacobs et al., 40% of immunocompromised patients with HRV-related respiratory symptoms were hospitalized, with 11% becoming critically ill [1]. Consequently, hospitalists and intensivists must maintain a high degree of clinical suspicion for respiratory decompensation in HRV-infected immunocompromised patients. In our patient’s case, we promptly intubated and admitted the patient to the intensive care unit (ICU), as we suspected ARDS early on in her disease course.

Rhinovirus as an etiology for the development of ARDS is exceedingly rare, only reported on a case report level, as can be seen in Table 2 [6-10]. Notably, most cases occurred in females and in patients who had chronic pulmonary diseases or patients who were immunosuppressed. Soni et al. report on a case of a female with HRV-related ARDS who had a history of asthma [6]. Hamid et al. report a case of a female with HRV-related ARDS who was on methotrexate therapy for rheumatoid arthritis [9]. Cecchini et al. also reported on a female patient with chronic obstructive pulmonary disease who developed HRV-related ARDS [10]. Thus, similar to these cases, we surmise that our patient’s major risk factors for the development of rhinovirus ARDS were a history of asthma and a history of chronic immunosuppressive therapy, namely budesonide and tacrolimus.

**Table 2:** Patient characteristics and oxygen therapy of patient cases in the relevant literature. HRV: human rhinovirus; ARDS: acute respiratory distress syndrome

Reference Number	Age	Sex	HRV Positive	Development of ARDS	Oxygen Therapy
[6]	22	Female	Yes - Nasopharyngeal Swab	Yes	Intubated
[7]	25	Female	Yes - Nasopharyngeal Swab	Yes	Intubated
[8]	59	Female	Yes - Nasopharyngeal Swab	Yes	High-Flow Nasal Cannula
[9]	70	Female	Yes - Nasopharyngeal Swab	Yes	Intubated
[10]	67	Male	Yes - Nasopharyngeal Swab	Yes	High-Flow Nasal Cannula

The treatment of HRV-induced ARDS is no different than ARDS caused by any other etiology - we followed a low tidal volume strategy of 6 ml/kg of ideal body weight to prevent barotrauma based on the Acute Respiratory Distress Syndrome Network (ARDSNet) study, as this has shown mortality benefit. ARDS is associated with poor lung compliance, and reducing lung stretching by employing a low tidal volume strategy has been shown to be efficacious in the management of patients with ARDS. Additionally, we aimed for a plateau pressure of less than 30 mmHg and driving pressure of less than 15 mmHg based on the ARDSNet protocol [11]. We believe that the implementation of this strategy contributed to the patient's rapid recovery, and we recommend that clinicians continue to employ this approach when managing HRV-induced ARDS cases.

This patient case is unique in that it demonstrates a rare case of ARDS caused by rhinovirus infection. Additionally, similar to the currently limited evidence available, our patient was immunosuppressed and had a history of chronic pulmonary disease, increasing her risk of susceptibility to HRV infection and developing more severe manifestations of HRV infections. When encountering immunosuppressed patients with respiratory distress, it is important for clinicians to consider HRV as a potential etiology and be prepared to escalate care promptly, as these patients may progress to developing ARDS. The ARDSNet protocol of low tidal volume mechanical ventilation strategy should be employed, as in any other case of ARDS. Future studies may shed light on gender-related predisposition to HRV-related ARDS and explore other pharmacologic agents that may be useful in immunosuppressed patients who develop this condition.

## Conclusions

Human rhinovirus seldom leads to community-acquired pneumonia in immunocompetent hosts, and it is even rare for it to progress to acute respiratory distress syndrome requiring mechanical ventilation. In immunosuppressed patients, HRV may lead to community-acquired pneumonia, but rarely does it ever lead to ARDS. We describe the management of an immunosuppressed patient who was diagnosed with human rhinovirus pneumonia complicated by ARDS. She was promptly managed with lung protective ventilation strategies and made a rapid recovery. We aim to increase awareness of this rare manifestation of rhinovirus infections, as it is easily transmissible and may be devastating to immunocompromised hosts.
